# Evolution of national and European policies in the field of rare diseases and their impact over the past five years

**DOI:** 10.1186/1750-1172-9-S1-P13

**Published:** 2014-11-11

**Authors:** Charlotte Rodwell, Ségolène Aymé

**Affiliations:** 1 EUCERD Scientific Secretariat, INSERM, US14, Orphanet, France

## 

**Figure 1:**
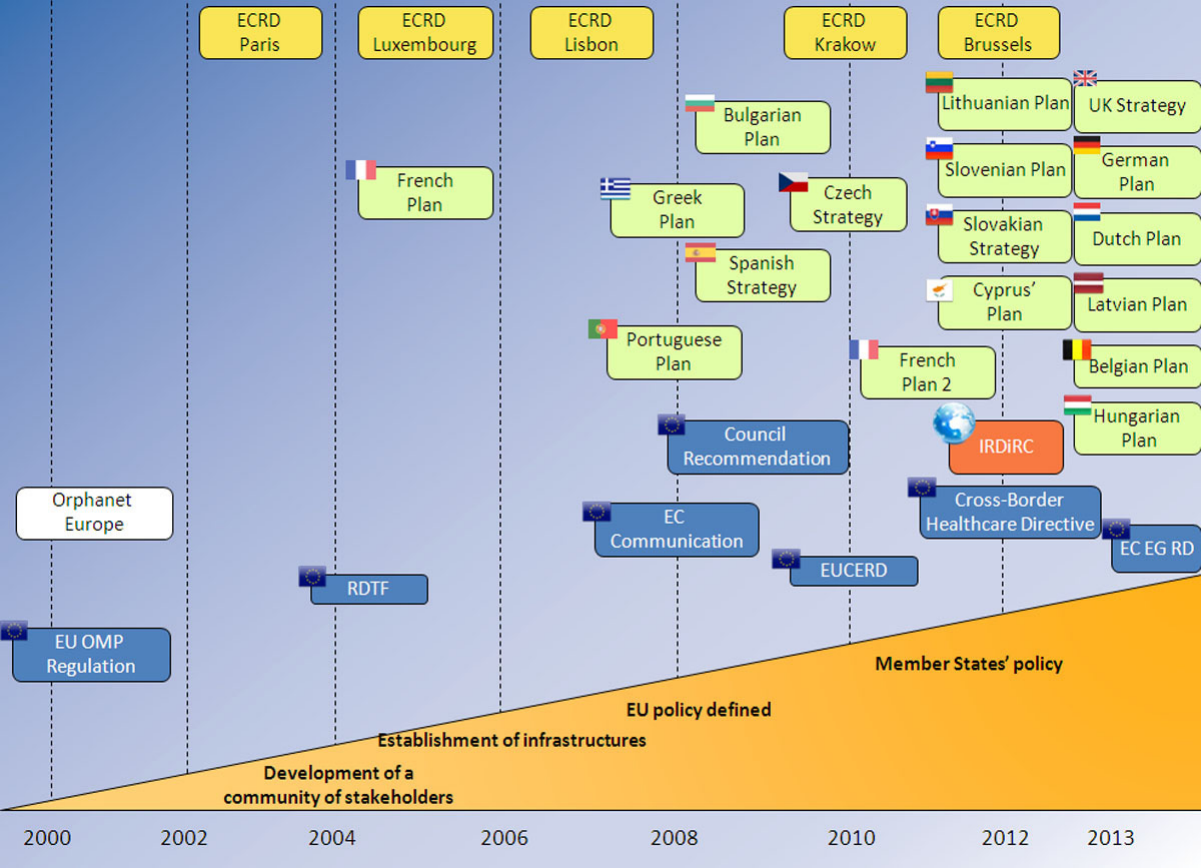
Emergence of concepts and initiatives surrounding rare diseases in Europe (December 2013)

**Figure 2:**
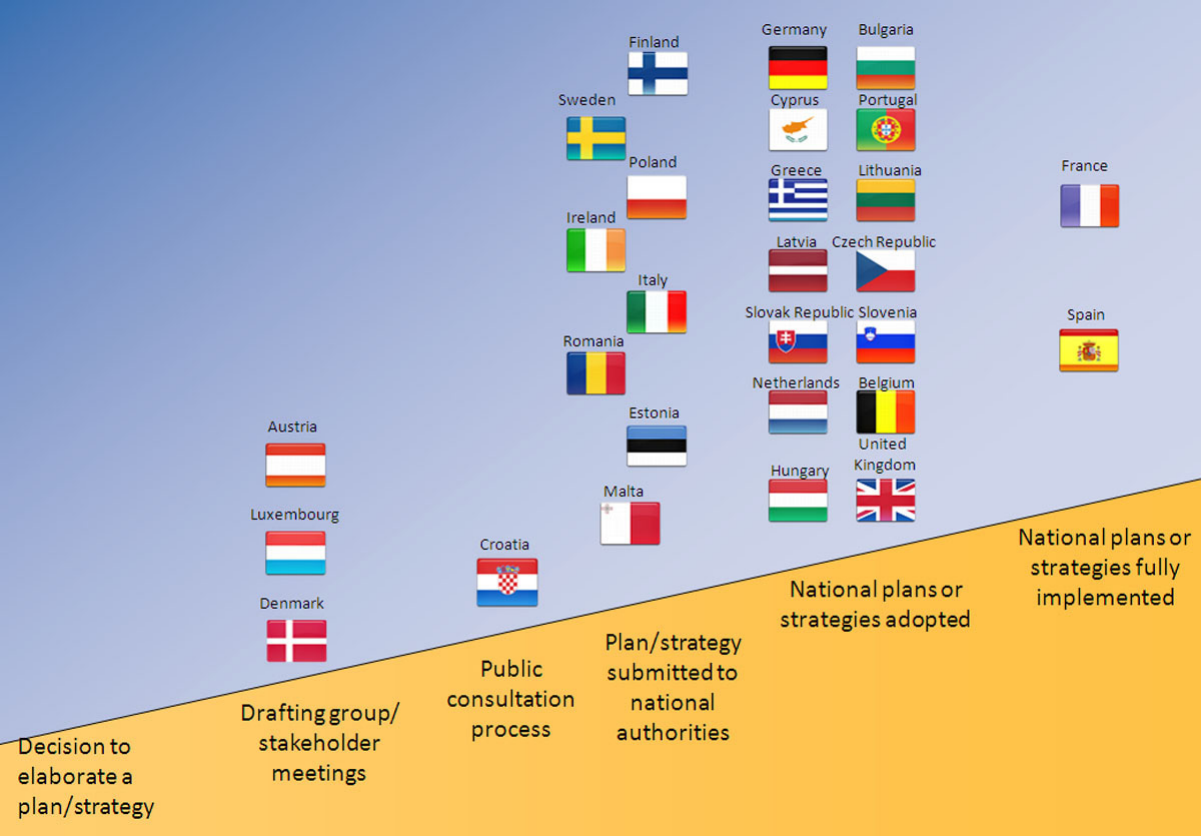
Stages of development of national plans or strategies for rare diseases in EU MS in December 2013

The 2009 *Council Recommendation on an Action in the Field of Rare Diseases* (*2009/C 151/02*) [1] encouraged Member States to elaborate a national plan or strategy for rare diseases before the end of 2013. One of the principal tasks of the European Union Committee of Experts on Rare Diseases (EUCERD) [2], through its Scientific Secretariat, was to analyse the results of the actions cited in the Recommendation, notably through the production of the annual report on the ‘State of the Art of Rare Disease Activities in Europe’ [3]. The 2014 edition of the report considers the impact of European policy and national plans/strategies (Figure 1) on the organisation of health care and services for patients with rare diseases, including access to diagnosis and treatment, as well as considering progress in the field of registries, rare disease research initiatives, the genetic testing offer, access to information on rare diseases, patient organisations, and specialised social services. In terms of national plans and strategies for rare diseases, by end 2013, the deadline to elaborate national plans/strategies for rare diseases fixed by the Council Recommendation, most EU Member States had submitted a plan/strategy to their national authorities and sixteen countries have adopted a plan/strategy (Figure 2). France and Spain have implemented and assessed their first plan. As their number one priority, most countries plan to identify and design centres of expertise for rare diseases. Many of these plans/strategies, however, have no dedicated budget for their actions, a result of the unfavourable economic context which may hinder the implementation of defined measures. The next challenge for EU Member States will be to effectively implement and assess these plans, which the new Commission Expert Group on Rare Diseases [4] will follow closely. Research in the field of rare diseases has been boosted in recent years due to European and international initiatives, such as European research projects funded via the DG Research and Innovation framework programmes and the E-Rare ERA-NET [5] as well as the creation of the International Rare Disease Research Consortium (IRDIRC) [6]. The Orphanet database [7] continues to expand and provides data collected in 37 European and surrounding countries with partner teams in Canada and Australia and negotiations ongoing in other world regions. Nearly all countries now have a national alliance for rare diseases ensuring that the voice of rare diseases, carried by Eurordis [8] at the European level, is heard.
